# Canaloplasty versus Nonpenetrating Deep Sclerectomy: 2-Year Results and Quality of Life Assessment

**DOI:** 10.1155/2018/2347593

**Published:** 2018-02-25

**Authors:** Anna Byszewska, Anselm Jünemann, Marek Rękas

**Affiliations:** ^1^Department of Ophthalmology, Military Institute of Medicine, Warsaw, Poland; ^2^Department of Ophthalmology, University Eye Hospital, Rostock, Germany

## Abstract

**Purpose:**

To compare phacocanaloplasty (PC) and phaco-non-penetrating deep sclerectomy (PDS).

**Methods:**

75 patients with uncontrolled glaucoma and cataract were randomized for PC (37 eyes) or PDS (38 eyes). Intraocular pressure (IOP) and number of medications (meds) were prospectively evaluated. Follow-up examinations were performed on days 1 and 7 and after 1, 3, 6, 12, 18, and 24 months. Surgical success was calculated. Complications and postoperative interventions were noted. Quality of life (QoL) was analyzed.

**Results:**

Preoperatively, mean IOP and meds were comparable (*P* > 0.05). After 24 months, IOP significantly decreased in PC from 19.4 ± 5.9 mmHg (2.6 ± 0.9 meds) to 13.8 ± 3.3 mmHg (0.5 ± 0.9 meds) and in PDS from 19.7 ± 5.4 mmHg (2.9 ± 0.9 meds) to 15.1 ± 2.9 mmHg (1.1 ± 1.2 meds). Statistically lower IOP was observed in PC in the 6th month and persisted until 24 months (*P* < 0.05). No difference was found in meds (except for month 18, in which less drugs were used in PC (*P* = 0.001)) or success rates (*P* > 0.05). The most frequent complication in PC was transient hyphema (46%), in PDS bleb fibrosis (24%). PC patients during postoperative period required only goniopuncture (22% of subjects), whereas PDS patients required, in order to maintain subconjuctival outflow, subconjunctival 5-fluorouracil injections in 95% of cases (median = 3), suture lysis (34%), needling (24%), and goniopuncture (37%). NEI VFQ-25 mean composite score for PC was 78.04 ± 24.36 points and for PDS 74.29 ± 24.45 (*P* = 0.136). *α* Cronbach's correlation coefficient was 0.913.

**Conclusions:**

PC leads to a more effective decrease in IOP than PDS in midterm observation with similar safety profiles. PDS patients required a vast number of additional procedures in contrast to PC patients, but this fact did not influence QoL.

## 1. Introduction

The main aim of this study is to compare canaloplasty to other nonpenetrating procedures in terms of safety, efficacy, and postoperative quality of life. It is a continuation of a previously published 12-month observation [1]. However, the outcomes changed as the studied population grew, due to higher statistical power.

Canaloplasty is a relatively new procedure, as it was introduced in 2007 by Lewis et al. [2]. To date, canaloplasty has already been on the market for 10 years. Although it requires surgical skills at a certain high level and is technically challenging, it has aroused growing interest among glaucoma surgeons, which has resulted in numerous papers published on the subject. Also, various modifications of the classic procedures were proposed, yet not all published but presented at congresses such as canaloplasty ab interno [3, 4], canaloplasty with suprachoroidal drainage [5], or with an alternative system for intubation without viscodilation [6]. This procedure is very appealing to glaucoma surgeons, especially because it boosts the anatomy and physiology of the eye and restores natural outflow pathways of aqueous humor [2].

To date, there are few published papers comparing canaloplasty to other surgical procedures, and in those few available, it is being compared to trabeculectomy, to viscocanalostomy, or to Hydrus. We chose sclerectomy as for comparison, because the classic canaloplasty surgical technique stems from nonpenetrating deep sclerectomy. Over the course of both procedures, the anterior chamber does not communicate directly with the scleral wound and the outflow is due to perlocation of the aqueous humor through trabeculo-Descemet membrane (TDM), among other outflow pathways. This fact supports the equal standing of deep sclerectomy and canaloplasty, especially regarding overfiltration risk and complications associated with it, such as hypotonic maculopathy, shallow anterior chamber, or choroidal effusion [7].

Nowadays, much attention is paid to quality of life [8–12]. According to EGS guidelines, the goal in glaucoma treatment is not only to maintain visual function, but also related to QoL at a sustainable cost. On the grounds of differences in postoperative care, patients were asked questions associated with QoL. The goal was to assess whether additional interventions associated with filtering bleb, such as needling or 5-fluorouracil (5-FU) injections, greater number of follow-up visits indeed influence patients' QoL.

## 2. Materials and Methods

Patients and methods were previously described, as this paper presents further observation of already published data [1].

### 2.1. Patients

The tenets of the World Medical Association Declaration of Helsinki and the principles developed by the European Union entitled Good Clinical Practice for Trials on Medical Products in the European Community were followed in this study. The project was approved by the Bioethics Committee of the Military Institute of Medicine in Warsaw. The study was registered at NCT01726543.

The inclusion criteria were coexisting glaucoma and cataract (NC1 and NC2) classified according to the LOCS III scale. Glaucoma types included were primary open-angle glaucoma (POAG) and pseudoexfoliation glaucoma (PEX). Written consent was obtained from all of the participants after detailed explanation of the procedure and surgical alternatives and after declaring their willingness to participate in the study.

Enrollment into groups was carried out by a random sorting algorithm with an allocation ratio set to 1.0 on the day of surgery.

Preoperative examination, randomization, and postoperative care were performed by single physician (first author—Anna Byszewska), who had no interest in setting one procedure in favor of another. Surgeon was excluded from any medical activities in order to avoid bias.

### 2.2. Preoperative Examination

Preoperatively, general and ophthalmic medical history was taken. Baseline examination included intraocular pressure (IOP), uncorrected distance visual acuity, best-corrected distance visual acuity (BCVA), and slit lamp examination of the anterior and posterior segments of the eye. In addition, central corneal thickness (CCT), axial length (AXL), keratometric parameters, required for IOL calculation, were measured, and gonioscopy was performed. IOP was measured during the preoperative visit as a diurnal curve and on the day of surgery as a single measurement between 8 and 10 am. IOL was calculated on the basis of the SRK T formula.

### 2.3. Surgical Technique

Surgical techniques were described in detail in a previously published paper [1]. All surgical procedures were performed under retrobulbar anaesthesia (2% xylocaine and 0.5% bupivacaine) by one surgeon (M.R.). Classic canaloplasty was carried out with a standard canaloplasty set (iTrack from Ellex Medical Lasers Pty Ltd., Adelaide, Australia). Nonpenetrating deep sclerectomy was carried out with Healaflow implant, a slowly resorbable crosslinked viscoelastic gel (Anteis Ophthalmology, Geneva, Switzerland).

In both procedures, fornix-based superficial scleral flap was dissected, followed by deep scleral flap and TDM dissection. During the next step, a 2.2 mm clear corneal temporal incision was made, the cataract was phacoemulsified (Infiniti Vision System, Alcon Surgical, Fort Worth, TX), and an IOL was implanted. The deep scleral flap was excised.

In PC, 360° of Schlemm's canal circumference was catheterized and viscodilated with prolene suture left under tension to distend the trabecular meshwork inward. In PDS, after dissection of TDM, the roof of Schlemm's canal was removed. The superficial scleral flap was then loosely sutured to the sclera, and HealaFlow was injected under the flap to create a filtering bleb. In PC, the superficial flap was sutured tightly in order to prevent leakage and subsequent bleb formation with interrupted 10–0 monofilament nylon suture. The conjunctiva was sutured down over the limbus with one interrupted 6.0 Vicryl suture.

### 2.4. Postoperative Protocol

The minimum scheme of postoperative visits included days 1 and 7 and 1, 3, 6, 12, 18, and 24 months after surgery. All patients received a topical steroid and antibiotic combination for 4 weeks after surgery.

During follow-up examinations, BCVA was determined with an EDTRS chart, and IOP was measured with a Goldmann applanation tonometer. All IOP measurements included in analysis were taken between 8 and 10 am. The anterior segment and fundus were examined. Number of hypotensive medications was noted. In PC, gonioscopy was additionally carried out to detect any complications associated with this procedure.

On the basis of single IOP measurements, the course of mean IOP and percentage reduction from baseline were calculated. Surgical success was analyzed in two categories, complete and qualified, and was performed by the Kaplan-Meier method. Complete surgical success was defined as IOP ≤ 18 mmHg with no antiglaucoma medications, and qualified success was defined as IOP ≤ 18 mmHg with or without medications. A procedure was considered to be a failure when IOP was >18 mmHg or when an eye required further glaucoma drainage surgery. On the day of surgery, glaucoma drugs were discontinued. When surgery was unsuccessful, medications were administered again in accordance with the guidelines of the European Glaucoma Society.

All complications were noted, and those, which occurred within 30 days following surgery, were considered early, whereas after 30 days were counted as late. Additionally, procedures which were carried out to maintain IOP at a sufficiently low level were noted. These included, for PDS, 5-FU subconjunctival injections (when signs of bleb failure were noticed, such as new, tortuous vessels, and hyperemia at filtering bleb or encapsulation), suture lysis (performed within 14 days with argon laser, when tight sutures on deep scleral flap prevented subconjunctival filtration), and needling (in case of encapsulated and flat blebs, which caused elevated IOP). Goniopuncture was performed in both groups when filtration through TDM was suspected to be insufficient accompanied by elevated IOP. The indication for all additional procedures was insufficient IOP reduction accompanied by morphological changes at surgical site mentioned above. 5-Fluorouracil in a dose of 0.2 ml (5 mg) was injected into the subconjunctival space 180° from the sclerectomy site. Needling of filtering bleb was performed in biomicroscopy, following proxymetacaine eye drop instillation. 5-FU was usually applied after needling. During goniopuncture with an Nd:YAG laser, three to 20 shots were applied using energy ranging from 2 to 4 mJ.

Visual field (VF) examination was performed with Humphrey Field Analyzer (Carl Zeiss Meditec AG, Germany) with 24-2 testing algorithm, prior to surgery, then after 12 and 24 months in order to assess the rate of glaucoma progression. Only VF with fixation losses <20% and summary reliability indices (false responses and fixation losses) not exceeding 30% were taken for analysis. Mean deviation (MD) and pattern standard deviation (PSD) were analyzed.

To assess postoperative quality of life, patients were asked to complete a questionnaire which consisted of the National Eye Institute Visual Function Questionnaire 25 (NEI VFQ-25) [13]. The questionnaire was not included in the study from the beginning and that is why answers from 22 consecutive patients from each group (44 all together) were obtained. The answers were given between the 3rd and 6th month following the surgery. Patients received the questionnaire during the 3rd month of follow-up and self-administered it at home.

A Polish version was used in this study. Great care was taken to produce an accurate translation of the original form, according to standardized methodology used for QoL questionnaires [14]. It was first translated into Polish by a native speaker, then back to English. Afterwards, it was revised by clinicians for better accuracy.

NEI VFQ-25 measures the influence of visual impairment on various aspects of life [13, 15]. Questions address three main categories: general health, quality of vision, and vision-related quality of life (VR-QoL). Within those three areas, twelve scales (groups of questions or subscales) are presented as follows: general health, general vision, near vision, distance vision, driving, peripheral vision, color vision, ocular pain, role limitations, dependency, social functions, and mental health [13]. Each subscale consists of minimum one item and maximum of 4 items. In order to calculate the scores, the standard algorithm was used. Possible scores after conversion range from 0 to 100, where higher scores represent better quality of life and visual function. To calculate the composite score, the general health scale was excluded and the average of 11 scale scores was calculated.

### 2.5. Statistical Analysis

Statistical analysis of the investigated variables was performed. The Shapiro-Wilk test was used to assess the compliance of the parameters with normal distribution. For one-way analysis of variance (ANOVA) purposes, the post hoc Bonferroni test was used in multiple comparisons or the Kruskal-Wallis test in the case of noncompliance of the parameters with normal distribution. The analysis between the groups was performed using Mann–Whitney *U* test. The frequency table and Chi-squared test were used for comparing quality characteristics. The Kaplan-Meier method was used for calculation of survival plots, and the differences were tested with the log-rank test. Apart from standard statistical methods, analysis of the questionnaire included the Cronbach's *α* coefficient calculation. The significance level *P* < 0.05 was adopted for the purposes of calculations. Calculations were performed in Statistica 10.0 PL software.

## 3. Results

Data was obtained from 75 patients, of whom 37 underwent PC and 38 PDS. The mean follow-up period taken for statistical analysis was 22.3 ± 5.1 months. Details of patients' demographic data are summarized in Table 1.

The final 24-month data was gathered from 30 PC and 34 PDS patients (Table 2).

The grounds for preterm terminations were as follows: 3 patients died due to unrelated reasons (after 1st, 12th, and 18th month of follow-up). One PDS patient resigned from the study after central retinal vein occlusion development 3 months after surgery. Three patients were lost after 18 and two after 12 months. Another two resigned because of personal reasons after 3 and 6 months.

### 3.1. Control of Intraocular Pressure

**Figure 1:**
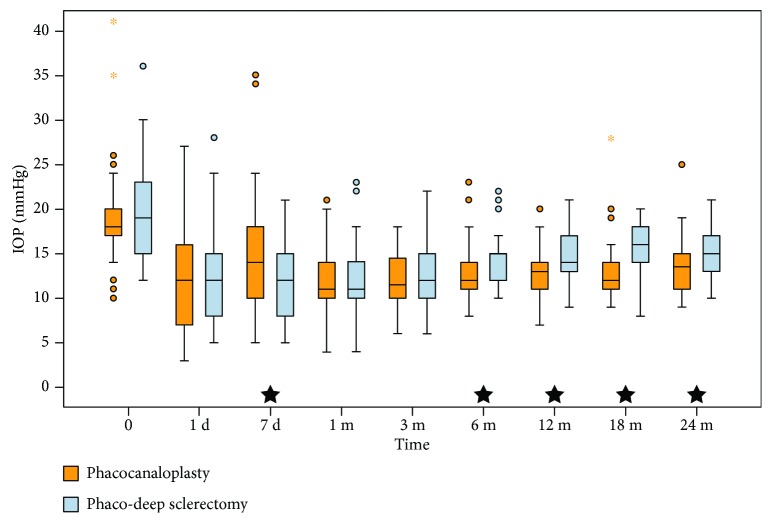
Box plot with intraocular pressure values. Black star at the bottom indicates statistical difference between the groups.

The IOP was well controlled in both groups (Figure 1). Mean baseline IOP in PC was 19.4 ± 5.8 mmHg and 19.7 ± 5.4 mmHg in PDS and did not differ between the groups (*P* = 0.639). At the end of observation, mean IOP significantly decreased to 13.8 ± 3.3 mmHg (*P* = 0.001) and 15.1 ± 2.9 mmHg (*P* = 0.001), respectively (Table 2). Mean IOP was reduced by 25.7% in PC and 18.9% in PDS. Mean IOP was lower for PDS on the 7th day postop (*P* = 0.023). There was no difference in the 1st and 3rd months of follow-up. Starting from the 6th month, mean IOP was lower in PC, and the difference lasted until 24 months (*P* = 0.048).

### 3.2. Medications

**Figure 2:**
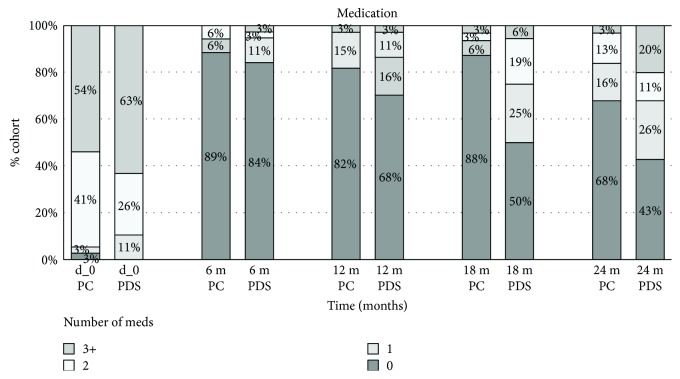
Medications in phacocanaloplasty and phaco-deep sclerectomy.

Fewer medications were used after surgery than before in both groups (*P* < 0.05). Baseline mean number of meds in PC was 2.6 ± 0.9, Me = 3.0, and in PDS, 2.9 ± 0.9, Me = 3.0 (*P* = 0.197). The number of meds significantly decreased postoperatively. At 12 months, the mean number of hypotensive drugs for both surgeries was zero. The number of medications used grew over time in both groups but faster in the PDS group, to reach a mean of 0.5 ± 0.9 meds in PC and 1.1 ± 1.2 meds in PDS. No differences were noticed throughout the study, except for month 18, in which less drugs were used in PC (*P* = 0.001). No further differences were observed over the course of the 24-month follow-up (*P* = 0.058). However, two years after surgery, median in PC was zero, whereas in PDS, it was 1 drug (Table 2). At 24 months, 68% of PC and 43% of PDS did not require any antiglaucoma medication (Figure 2).

### 3.3. Surgical Success

**Figure 3:**
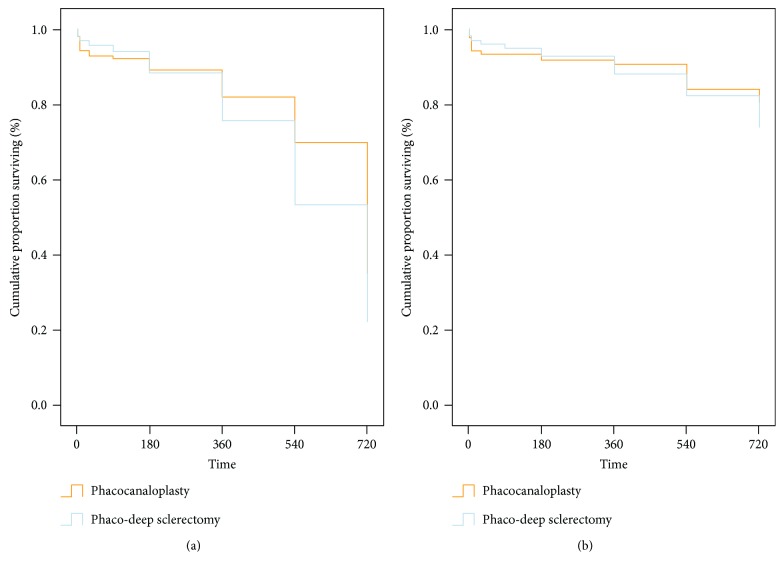
Plot depicting cumulated percentage of survival rates for criterion ≤18 mmHg. (a) Qualified success; (b) complete success.

The proportion of operated eyes meeting the 18 mmHg criterion for the qualified success rate after 24 months of follow-up was 80.5% of the PC group and 73.8% of PDS (chi^2^ = 0.03, df = 1, *P* = 0.957) and is presented in Table 3. Complete success rates were, respectively, 34.9% and 22.1% (chi^2^ = 2.22, df = 1, *P* = 0.136). No differences in surgical success were observed during the study. In the first 6 months, PDS was characterized by less failures. After 6 months, the higher survival percentage was in favor of PC patients and this trend lasted until the 24th month. The complete success survival plot lies high and is relatively parallel to the *x*-axis, which is typical for a low failure rate. Use of antiglaucoma meds shifts the plot even higher, which means good control of IOP with hypotensive drops (Figure 3).

### 3.4. Corrected Distance Visual Acuity

**Figure 4:**
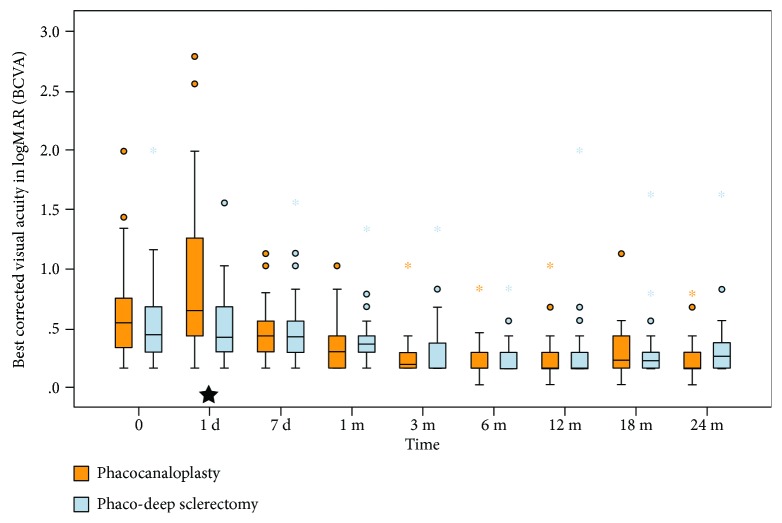
Box plot of BCVA in logMAR during a 24-month period. Black star at the bottom indicates statistical difference between the groups.

In the analyzed period, mean BCVA improved significantly in the PC group from 0.40 ± 0.43 to 0.05 ± 0.12 logMAR and in PDS from 0.30 ± 0.32 to 0.12 ± 0.23 logMAR (*P* < 0.001 for both groups). At baseline, no differences were noted (*P* = 0.314). On the 1st postop day, the mean BCVA was better in PDS (*P* = 0.07); however, no differences were found in further observation (Figure 4).

Two years after surgery, stable vision or improvement of one or more Snellen lines was present in all patients after PC and in the majority of PDS (85.3%). A decline of 1 line was noted in 3 patients after PDS (8.8%). Decline of 2 lines was observed in 2 subjects (5.9%). One of them developed diabetic macular edema, and the second had transient visual acuity instability, as good vision was regained at the following examination (0.0 logMAR). No changes in BCVA were observed in 8.8% PDS and 13.3% PC eyes.

### 3.5. Complications and Additional Procedures

The most frequent early postoperative complication in the PC group was hyphema, which was observed in 17 eyes (46%) and microhyphema noted in 10 eyes (27%). All cases of hyphema, but for one, resolved spontaneously within 2 weeks. One PC eye underwent anterior chamber lavage in the early postoperative period to remove hyphema. Peripheral Descemet membrane detachment was noted in one PC case, and it also resolved spontaneously with no influence on VA. In the PDS group, bleb fibrosis was found in 9 cases (24%). Complication rates are summarized in Table 4.

Late complications included two iris incarcerations in PDS patients. One was observed in the 18th month of follow-up. The patient suffered from atopic dermatitis and often rubbed his eye, which caused incarceration of the iris and changed the pupil's shape. This patient was administered with antiglaucoma drops and did not require surgical intervention. The second one had iris incarceration following late goniopuncture. During postgoniopuncture follow-up visit, distortion of pupil and IOP increase was noticed and the eye did not respond to topical drops and was reoperated. One of PDS developed central retinal vein occlusion between the 1st and 3rd postoperative visits.

In PDS, 36 (94.7%) eyes received 5-FU injections. The mean number of 5-FU injections per patient was 3.7 (ME = 3) and ranged from 1 to 10. Nd:YAG laser goniopuncture was performed in 14 (36.6%) eyes, suture lysis in 13 (34%), and needling in 9 (24%) of the operated eyes. Goniopuncture was the only eligible intervention for PC and was done in 8 (22%) subjects. The data is presented in Table 4.

### 3.6. Visual Fields

Complete set (before 12 and 24 months) of reliable visual fields was available from 35 PC and 29 PDS patients. Preoperatively, mean MD in case of PC was MD_0 = −8.80 ± 7.57 dB (ME = −5.05 dB) whereas in PDS, MD_0 = −7.16 ± 6.79 dB (ME = −4.72 dB) and did not differ between groups (ME = −5.18 dB). Twelve months after the procedure, respectively, MD_12 = −8.63 ± 7.84 dB (ME = −5.05 dB) and MD_12 = −6.11 ± 6.41 dB (ME = −4.72 dB) (*P* = 0.418). Two years after the procedure MD_24 = −7.48 ± 7.21 dB (ME = −5.18 dB) and in the PSD MD_24 = −5.95 ± 6.54 dB (ME = −4.02) (*P* = 0.477). There were no statistically significant differences in MD between the groups during the follow-up period. The course of the MD parameter was not stable throughout the observation. In PC, its value becomes less negative than before the surgery (Friedman ANOVA, *P* = 0.008). Wilcoxon rank post hoc test showed less negative MD 12 months after surgery compared to baseline (*P* = 0.016). Also after 2 years, MD was less negative than before surgery (*P* = 0.031). However, there was no difference between MD one and two years after surgery (*P* = 0.522).

In PDS, MD was stable throughout the follow-up period and no statistical differences were found in the individual observation periods (Friedman ANOVA, *P* = 0.152).

PSD was stable throughout the follow-up period in both study groups, and no differences were found between groups in any time point (*P* > 0.05). The mean PSD parameter for PC was about 6 dB (Friedman ANOVA *P* = 0.496) and about 3 dB in PDS (Friedman ANOVA *P* = 0.316).

### 3.7. Quality of Life

The composite score of NEI VFQ-25 was 77 points in both groups, whereas separately, the mean score for PC was 78.04 Me = 85 and for PDS 74.29 points Me = 79. There were no differences in mean composite scores between the groups (*P* = 0.136). All scales concerning postoperative general health, general vision, eye pain, near vision, distant vision, peripheral field, social functioning, color vision, driving, role limitations, mental health, and dependency were analyzed separately for both groups. None of the obtained subscale scores differed between groups. The NEI VFQ-25 scores are presented in Table 5.

The alpha Cronbach's correlation coefficient was 0.913 for both groups, while separately 0.929 for PC and 0.919 for PDS. No differences were noted in Cronbach's alpha coefficients (*P* > 0.05).

## 4. Discussion

Both surgeries are safe and effective in 24-month observation. IOP results are in line with papers describing both procedures separately [16, 17].

When the course of IOP is analyzed, the mean IOP was higher in PC at day 7, and the difference was statistically significant (*P* = 0.023). This could be due to the fact that it takes longer in canaloplasty to adjust to newly created anatomical outflow pathways, especially when Schlemm's canal or collector channels are collapsed [18, 19]. Some authors observe IOP spikes in the early postoperative period, which did not concern patients from this study. From the beginning, there was a slight but progressive rise in mean IOP in PDS, and this trend persisted until the end of observation. In contrast, the mean IOP in PC was more stable (Figure 1). What should be emphasised is the statistically lower mean IOP in favor of canaloplasty starting from the 6th month, which persisted until the 24th month. This was actually the main aim of this study, to learn whether there would be a difference in efficacy between both of these nonpenetrating procedures. From our data, it can be clearly concluded that indeed there is a difference in hypotensive effect.

Based on Kaplan-Meier cumulative probability survival plots, using the 18 mmHg criterion, plots can be described as relatively flat with a low number of failures. PC is characterized by more failures in the first six months, but after this time, the PC survival plot is much more stable. PC gains an advantage over PDS in rates of patients meeting the 18 mmHg criterion. Simultaneously, in PDS, a progressive decrease in survival probability is observed. Such a decrease is typical for bleb-dependent procedures and is mainly influenced by fibrotic processes.

Also, the number of antiglaucoma medications used in PDS rises faster over time. For both procedures, the median number of drops after a year is zero; however, in PDS, after 24 months, it reaches 1, whereas it is still zero in PC. Nonetheless, no statistical differences were found in the number of antiglaucoma medications, apart from the month 18 follow-up, where it was lower in PC. But there was no difference again in the 24th month.

BCVA was significantly worse during the first postoperative day in PC. This is mainly due to hyphemas which occurred intraoperatively or in the early postoperative period. No more difference in BCVA was observed on day 7, which proves fast visual recovery in PC.

A relatively high percentage of hyphemas and microhyphemas was observed in PC. This could be due to low baseline IOP, which may indicate patency of Schlemm's canal and opened collector channels. This may result in increased blood reflux during PC [18, 20, 21]. On the other hand, hyphema is a positive prognostic factor of surgical success [22].

In PDS visual field assessment, MD was stable throughout the follow-up period, but the mean MD increased compared to the presurgery. In PC, these changes were already statistically significant. The MD increase even after two years can be explained by cataract extraction, which has been shown to reduce the mean retinal sensitivity, as demonstrated in the AGIS studies [23]. In this paper, same as in the present study, MD improvement was achieved after combined operations, resulting in a better overall retinal sensitivity, which was decreased by cataract. In contrast, the PSD in the AGIS study remained stable, which was also observed in the study group. The increase in the MD parameter after cataract removal has also been demonstrated in other publications [24, 25].

The choice of quality of life questionnaire was very difficult, because even though many different question forms specific to glaucoma are available, there are none that would precisely measure discrepancies in glaucoma postsurgical quality of life. The NEI VFQ-25 questionnaire was chosen because it is known to be responsive [10] and repeatable [13] in glaucoma patients and was previously used in various glaucoma studies, for example in the Early Manifest Glaucoma Study [11], where the influence of newly detected glaucoma on QoL was measured. Medically (but nonsurgically) treated patients were compared to the observed groups. Generally, results suggest that the absence or delay of treatment did not influence vision-targeted QoL. However, lower scores were associated with reduced visual acuity or worse perimetric mean deviation (MD), regardless of the studied group [11]. Whereas, Jampel et al. [26] studied whether glaucoma and glaucoma suspect patients' QoL correlate with Esterman binocular visual field indices and other visual function tests, and they did not demonstrate a correlation. On the other hand, Blumberg et al. [27] showed correlation between 24 and 2 and even stronger correlation between 10 and 2 visual field and composite score of NEI VFQ-25. While Jones et al. [28] found that there is stable loss of QoL until MD = −5 dB (mean deviation), a rapid deterioration of QoL is present once visual field MD in better eye worsens beyond −15 dB.

Guedes et al. [29] compared medical versus surgical treatments of glaucoma with NEI VFQ-25, and no differences were found between the studied groups. Kotecha et al. [30] reported long-term QoL results in trabeculectomy versus tube studies, where surgery did not worsen QoL, which was stable over 5 years of observation, and no differences were noted between the groups at any time point. Pahlitzsch et al. [9], in their very recent paper, did not demonstrate a difference in QoL of patients operated with microinvasive glaucoma surgery (MIGS) and traditional trabeculectomy 6 months after surgery. Matlach et al. also could not find the right questionnaire to compare trabeculectomy and canaloplasty and made their own, choosing selected questions from a variety of popular literature QoL question forms [8]. In their survey, the expected difference in QoL was obtained.

In our data, the answers in the NEI VFQ-25 questionnaire were highly consistent with Cronbach's *α* correlation coefficient 0.92, which is high, showing that the answers did correlate well with each other. Nonetheless, it showed no difference between the groups in either of the studied areas. It is a fact that in PC less procedures are carried out postoperatively in comparison to bleb-dependent methods [8, 31, 32]. This could have a negative influence on quality of life in PDS.

The similarity of quality of life scores is somewhat disappointing, as the difference in QoL in the early postoperative period was expected to be the main distinctive factor of both surgeries. The most intensive postoperative care usually lasts until the first month. However, in some cases, it may last even three months, especially in PDS due to the necessity of filtering bleb maintenance. After this period, postoperative care for both procedures is equal. Thus, subjects in the study were asked to fill out the questionnaire between the 3rd and 6th months of follow-up. They remembered the visits and feelings associated with them well. Matlach et al. [8] chose to carry out the QoL study after a 24-month period. They compared QoL in canaloplasty and trabeculectomy, asked questions associated with surgical aspects, and found higher QoL scores and satisfaction regarding canaloplasty. On one hand, two years after surgery is too late to remember the initial postoperative period; however, a longer period is probably better for the overall assessment. On the other hand, the authors did not calculate the internal consistency of the test, and it was not validated, which could have an influence on the reliability of the final results.

Nonetheless, glaucoma specialists are aware of distinctions in quality and in the number of necessary postoperative visits, especially when one procedure is bleb-dependent and another is not, as noticed by Brüggeman et al. [31]. Based on our experience, many more PDS patients complained of superficial eye disease symptoms, most frequently associated with 5-FU injections. Brüggeman et al. compared trabeculectomy and canaloplasty in a 12-month observation of one patient and stated that IOP results did not differ significantly, but complication rates were much higher in traditional filtering surgery. In canaloplasty, there were no bleb management issues, much less interventions, which resulted in fewer postoperative visits. These conclusions are in line with our results. However, again, the questionnaire showed no difference.

The conclusion of the quality of life study is that either it is not such a big difference for the patient as we would presume whether they undergo a deep sclerectomy, a bleb-dependent procedure, or canaloplasty, as both of them are safe and effective or methods sensitive enough to show that the fine discrepancies are not available. Although the groups in the quality of life study are not numerous, there are some very important aspects—it was a prospective study, and patients were randomly assigned to groups. They did not differ preoperatively and were all operated by one experienced surgeon. Postoperative care was also given in the same facilities by the same experienced physician in all cases.

## 5. Conclusions

In prospective and randomized study, canaloplasty leads to a more effective decrease in IOP than nonpenetrating deep sclerectomy in midterm observation with similar safety profiles.

PDS patients require a vast number of additional procedures in contrast to PC patients. Nonetheless, this fact does not influence QoL measured with NEI VFQ-25. New and more precise methods of QoL evaluation are required, with a questionnaire specific to the postoperative period.

## Figures and Tables

**Table 1:** Patients' demographic data.

Data	Phacocanaloplasty	Phaco-deep sclerectomy	*P* value
	Mean ± SD ratio		
*N*	37	38	0.908^∗^
Age (years)	75.1 ± 8.1	73.6 ± 6.2	0.079^#^
Sex (female/male)	15/22	21/17	0.202^∗^
Eye (right/left)	15/22	17/21	0.713^∗^
Glaucoma type: POAG/PEX	27/10	34/4	0.124^∗^

^∗^chi2; ^#^Mann–Whitney *U*.

**Table 2:** Number of patients (*N*), mean IOP, standard deviation of IOP (SD), Wilcoxon *P* values comparing base IOP values and, at different time points, mean number of antiglaucoma medications with standard deviations (meds), Me (median of meds), and the last two columns present statistical values of *P* based on Mann–Whitney *U* test regarding IOP and medications. Statistical significance was when *P* < 0.05 and was denoted with asterisk (^∗^) where appropriate.

	Phacocanaloplasty					Phaco-deep sclerectomy					*P* value	
	*N*	IOP mean ± SD	IOP Wilcoxon ^∗^*P*	Meds mean ± SD	Meds Me	*N*	IOP mean ± SD	IOP Wilcoxon ^∗^*P*	Meds mean ± SD	Meds Me	IOP	Meds
Preop	37	19.4 ± 5.8		2.6 ± 0.9	3.0	38	19.7 ± 5.4		2.9 ± 0.9	3.0	0.639	0.197
1_d	37	12.1 ± 6.0	0.000	0.0 ± 0.0	0.0	38	12.1 ± 5.7	<0.001	0.0 ± 0.0	0.0	0.983	1.000
7_d	36	15.2 ± 6.8	0.002	0.2 ± 0.8	0.0	38	11.7 ± 4.3	<0.001	0.0 ± 0.0	0.0	0.023^∗^	0.149
1_m	36	11.5 ± 3.8	<0.001	0.1 ± 0.4	0.0	38	12.2 ± 4.0	<0.001	0.0 ± 0.0	0.0	0.735	0.143
3_m	36	11.8 ± 3.3	<0.001	0.0 ± 0.2	0.0	38	12.8 ± 3.6	<0.001	0.0 ± 0.3	0.0	0.330	0.985
6_m	34	13.0 ± 3.2	<0.001	0.2 ± 0.5	0.0	37	14.2 ± 3.0	<0.001	0.2 ± 0.6	0.0	0.042^∗^	0.639
12_m	33	13.0 ± 3.0	<0.001	0.2 ± 0.6	0.0	37	14.6 ± 3.0	<0.001	0.5 ± 0.9	0.0	0.037^∗^	0.218
18_m	30	13.3 ± 4.0	<0.001	0.2 ± 0.7	0.0	33	15.5 ± 3.0	<0.001	0.9 ± 1.0	1.0	0.001^∗^	0.001^∗^
24_m	30	13.8 ± 3.3	<0.001	0.5 ± 0.9	0.0	34	15.1 ± 3.0	<0.001	1.1 ± 1.2	1.0	0.048^∗^	0.058

**Table 3:** Complete and qualified surgical success as a percentage with criterion of IOP ≤ 18 mmHg.

Surgical success IOP ≤ 18 mmHg				
	Phacocanaloplasty		Phaco-deep sclerectomy	
Follow-up	Complete (%)	Qualified (%)	Complete (%)	Qualified (%)
6 m	89.2	91.8	88.4	92.9
12 m	82.0	90.7	75.6	88.1
18 m	69.8	84.0	53.3	82.3
24 m	34.9	80.5	22.1	73.8

**(a):** 

Complications	Phacocanaloplasty (*N* = 37)	Phaco-deep sclerectomy (*N* = 38)	*P* value
	%/*n*	%/*n*	
Hypotony < 7 d	35% (13)	34% (13)	0.933
Hypotony > 30 days	13% (5)	8% (3)	0.431
Flare in anterior chamber (AC)	8% (3)	3% (1)	0.291
Choroidal effusion	5% (2)	0	0.146
Bleb fibrosis	0	24% (9)	0.002
Hyphema	46% (17)	0	0.001
Erythrocytes in AC	27% (10)	0	0.001

**(b):** 

Additional procedures	Phacocanaloplasty	Phaco-deep sclerectomy	*P* value
	%/(*n*)	%/(*n*)	
5-FU subconjunctival injections	0	95% (36) 3.75/person	0.001
Suture lysis	0	34% (13)	0.001
Goniopuncture	22% (8)	37% (14)	0.295
Needling	0	24% (9)	0.001

**Table 5:** NEI VFQ-25 scores; *P* value based on Mann–Whitney *U* test.

NEI scales	Phacocanaloplasty mean scores ± SD	Phaco-deep sclerectomy mean scores ± SD	N questions	*P* value
Composite score	78.0 ± 24.3	74.2 ± 24.4	25	0.136
General health	52.8 ± 21.4	51.3 ± 20.6	1	0.943
General vision	67.2 ± 18.2	67.5 ± 19.0	1	0.991
Ocular pain	63.6 ± 28.5	61.3 ± 26.1	2	0.849
Near activities	80.9 ± 18.9	73.8 ± 22.7	3	0.348
Distant activities	82.0 ± 18.9	77.2 ± 19.8	3	0.492
Peripherals field	78.4 ± 25.9	80.6 ± 26.6	1	0.636
Social functioning	90.3 ± 23.4	81.8 ± 23.0	2	0.104
Color vision	94.3 ± 13.2	87.5 ± 20.0	1	0.245
Driving			2	0.020
Role limitations	73.1 ± 26.4	66.6 ± 25.9	2	0.383
Mental health	71.0 ± 30.2	65.3 ± 26.7	4	0.578
Dependency	81.2 ± 19.8	75.3 ± 20.5	3	0.531

## References

[1] Rękas M., Byszewska A., Petz K., Wierzbowska J., Jünemann A. (2015). Canaloplasty versus non-penetrating deep sclerectomy - a prospective, randomised study of the safety and efficacy of combined cataract and glaucoma surgery; 12-month follow-up. *Graefe's Archive for Clinical and Experimental Ophthalmology*.

[2] Lewis R. A., von Wolff K., Tetz M. (2007). Canaloplasty: circumferential viscodilation and tensioning of Schlemm’s canal using a flexible microcatheter for the treatment of open-angle glaucoma in adults: interim clinical study analysis. *Journal of Cataract and Refractive Surgery*.

[3] Körber N. (2017). Canaloplasty ab interno - a minimally invasive alternative. *Klinische Monatsblätter für Augenheilkunde*.

[4] Francis B. A., Akil H., Bert B. B. (2017). Ab interno Schlemm’s canal surgery. *Developments in Ophthalmology*.

[5] Seuthe A.-M., Januschowski K., Mariacher S. (2018). The effect of canaloplasty with suprachoroidal drainage combined with cataract surgery - 1-year results. *Acta Ophthalmologica*.

[6] Scharioth G. B. (2015). Risk of circumferential viscodilation in viscocanalostomy. *Journal of Cataract and Refractive Surgery*.

[7] Eldaly M. A., Bunce C., Elsheikha O. Z., Wormald R., Cochrane Eyes and Vision Group (2014). Non-penetrating filtration surgery versus trabeculectomy for open-angle glaucoma. *Cochrane Database of Systematic Reviews*.

[8] Matlach J., Sauer J., Körber N. (2014). Quality of life following glaucoma surgery: canaloplasty versus trabeculectomy. *Clinical Ophthalmology*.

[9] Pahlitzsch M., Klamann M. K. J., Pahlitzsch M.-L., Gonnermann J., Torun N., Bertelmann E. (2017). Is there a change in the quality of life comparing the micro-invasive glaucoma surgery (MIGS) and the filtration technique trabeculectomy in glaucoma patients?. *Graefe's Archive for Clinical and Experimental Ophthalmology*.

[10] Hyman L. G., Komaroff E., Heijl A., Bengtsson B., Leske M. C. (2005). Treatment and vision-related quality of life in the early manifest glaucoma trial. *Ophthalmology*.

[11] Peters D., Heijl A., Brenner L., Bengtsson B. (2015). Visual impairment and vision-related quality of life in the early manifest glaucoma trial after 20 years of follow-up. *Acta Ophthalmologica*.

[12] Quaranta L., Riva I., Gerardi C., Oddone F., Floriano I., Konstas A. G. P. (2016). Quality of life in glaucoma: a review of the literature. *Advances in Therapy*.

[13] Mangione C. M., Lee P. P., Gutierrez P. R. (2001). Development of the 25-item National Eye Institute Visual Function Questionnaire. *Archives of Ophthalmology*.

[14] Acquadro C., Conway K., Hareendran A., Aaronson N., European regulatory issues and quality of life assessment (ERIQA) group (2008). Literature review of methods to translate health-related quality of life questionnaires for use in multinational clinical trials. *Value in Health*.

[15] Mangione C. M., Lee P. P., Pitts J. (1998). Psychometric properties of the National Eye Institute Visual Function Questionnaire (NEI-VFQ). *Archives of Ophthalmology*.

[16] Lewis R. A., von Wolff K., Tetz M. (2009). Canaloplasty: circumferential viscodilation and tensioning of Schlemm canal using a flexible microcatheter for the treatment of open-angle glaucoma in adults. *Journal of Cataract & Refractive Surgery*.

[17] Mermoud A., Schnyder C. C., Sickenberg M., Chiou A. G., Hédiguer S. E., Faggioni R. (1999). Comparison of deep sclerectomy with collagen implant and trabeculectomy in open-angle glaucoma. *Journal of Cataract & Refractive Surgery*.

[18] Grieshaber M., Pienaar A., Olivier J., Stegmann R. (2009). Channelography: imaging of the aqueous outflow pathway with flexible microcatheter and fluorescein in canaloplasty. *Klinische Monatsblätter für Augenheilkunde*.

[19] Battista S. A., Lu Z., Hofmann S., Freddo T., Overby D. R., Gong H. (2008). Reduction of the available area for aqueous humor outflow and increase in meshwork herniations into collector channels following acute IOP elevation in bovine eyes. *Investigative Ophthalmology & Visual Science*.

[20] Grieshaber M. C., Pienaar A., Olivier J., Stegmann R. (2010). Clinical evaluation of the aqueous outflow system in primary open-angle glaucoma for canaloplasty. *Investigative Ophthalmology & Visual Science*.

[21] Saraswathy S., Tan J. C., Yu F. (2016). Aqueous angiography: real-time and physiologic aqueous humor outflow imaging. *PLoS One*.

[22] Grieshaber M. C., Schoetzau A., Flammer J., Orgül S. (2013). Postoperative microhyphema as a positive prognostic indicator in canaloplasty. *Acta Ophthalmologica*.

[23] Koucheki B., Nouri-Mahdavi K., Patel G., Gaasterland D., Caprioli J. (2004). Visual field changes after cataract extraction: the AGIS experience. *American Journal of Ophthalmology*.

[24] Smith S. D., Katz J., Quigley H. A. (1997). Effect of cataract extraction on the results of automated perimetry in glaucoma. *Archives of Ophthalmology*.

[25] Seol B. R., Jeoung J. W., Park K. H. (2016). Changes of visual-field global indices after cataract surgery in primary open-angle glaucoma patients. *Japanese Journal of Ophthalmology*.

[26] Jampel H. D., Schwartz A., Pollack I., Abrams D., Weiss H., Miller R. (2002). Glaucoma patients’ assessment of their visual function and quality of life. *Journal of Glaucoma*.

[27] Blumberg D. M., De Moraes C. G., Prager A. J. (2017). Association between undetected 10-2 visual field damage and vision-related quality of life in patients with glaucoma. *JAMA Ophthalmology*.

[28] Jones L., Bryan S. R., Crabb D. P. (2017). Gradually then suddenly? Decline in vision-related quality of life as glaucoma worsens. *Journal of Ophthalmology*.

[29] Guedes R. A., Guedes V. M., Freitas S. M., Chaoubah A. (2013). Quality of life of medically versus surgically treated glaucoma patients. *Journal of Glaucoma*.

[30] Kotecha A., Feuer W. J., Barton K., Gedde S. J., Tube versus trabeculectomy study group (2017). Quality of life in the tube versus trabeculectomy study. *American Journal of Ophthalmology*.

[31] Brüggemann A., Despouy J. T., Wegent A., Müller M. (2013). Intraindividual comparison of canaloplasty versus trabeculectomy with mitomycin C in a single-surgeon series. *Journal of Glaucoma*.

[32] Januschowski K., Leers S., Haus A., Szurman P., Seuthe A.-M., Boden K. T. (2016). Is trabeculectomy really superior to canaloplasty?. *Acta Ophthalmologica*.

